# Development of HIV-1 Rectal-Specific Microbicides and Colonic Tissue Evaluation

**DOI:** 10.1371/journal.pone.0102585

**Published:** 2014-07-15

**Authors:** Charlene S. Dezzutti, Julie Russo, Lin Wang, Kaleab Z. Abebe, Jie Li, David R. Friend, Ian M. McGowan, Lisa C. Rohan

**Affiliations:** 1 Magee-Womens Research Institute, Pittsburgh, Pennsylvania, United States of America; 2 Department Obstetrics, Gynecology, and Reproductive Sciences, University of Pittsburgh, Pittsburgh, Pennsylvania, United States of America; 3 Department of Medicine, Division of General Internal Medicine, University of Pittsburgh, Pittsburgh, Pennsylvania, United States of America; 4 CONRAD, Arlington, Virginia, United States of America; 5 Department of Medicine, Division of Gastroenterology, Hepatology, and Nutrition, University of Pittsburgh, Pittsburgh, Pennsylvania, United States of America; 6 Department of Pharmaceutical Sciences, University of Pittsburgh, Pittsburgh, Pennsylvania, United States of America; National HIV and Retrovirology Laboratories, Canada

## Abstract

The gastrointestinal tract is structurally and functionally different from the vagina. Thus, the paradigm of topical microbicide development and evaluation has evolved to include rectal microbicides (RMs). Our interest was to create unique RM formulations to safely and effectively deliver antiretroviral drugs to mucosal tissue. RMs were designed to include those that spread and coat all surfaces of the rectum and distal colon rapidly (liquid) and those that create a deformable, erodible barrier and remain localized at the administration site (gel). Tenofovir (TFV) (1%) was formulated as an aqueous thermoreversible fluid and a carbopol-based aqueous hydrogel. Lipid-based liquid and gel formulations were prepared for UC781 (0.1%) using isopropyl myristate and GTCC (Caprylic/Capric Triglycerides), respectively. Formulations were characterized for pH, viscosity, osmolality, and drug content. Pre-clinical testing incorporated ex vivo colonic tissue obtained through surgical resections and flexible sigmoidoscopy (flex sig). As this was the first time using tissue from both sources side-by-side, the ability to replicate HIV-1 was compared. Efficacy of the RM formulations was tested by applying the products with HIV-1 directly to polarized colonic tissue and following viral replication. Safety of the formulations was determined by MTT assay and histology. All products had a neutral pH and were isoosmolar. While HIV-1_BaL_ and HIV-1_JR-CSF_ alone and in the presence of semen had similar replication trends between surgically resected and flex sig tissues, the magnitude of viral replication was significantly better in flex sig tissues. Both TFV and UC781 formulations protected the colonic tissue, regardless of tissue source, from HIV-1 and retained tissue viability and architecture. Our in vitro and ex vivo results show successful formulation of unique RMs. Moreover, the results of flex sig and surgically resected tissues were comparable suggesting the incorporation of both in pre-clinical testing algorithms.

## Introduction

Globally, the primary spread of HIV-1 is through heterosexual sex and women bear the greatest burden of infections [1]. Early efforts focused on prevention modalities that women could use independently of their partner to protect themselves from infection. Thus, the paradigm for microbicide development and use was focused on vaginal products; gels women would use prior to coitus independent of their partners consent to prevent the acquisition of HIV-1 [2]. In developed countries, the epidemic is spread primarily among those who engage in receptive anal intercourse (RAI) [3]. Indeed, some areas of the United States have incidence rates among black men who have sex with men (MSM) that compare equally to those of the generalized HIV epidemic in sub-Saharan Africa [4] and of marginalized MSM populations in Asia and Africa [5]. Further, RAI has been an underappreciated activity among heterosexual couples worldwide and may contribute to the HIV-1 epidemic more than previously thought [6]–[8]. Because of this, vaginal microbicides when developed would be used by all persons wanting to protect themselves from HIV-1 infection and would likely be used rectally. However, the structure (luminal area, epithelium, and immune cell numbers) and environment (pH and flora) of the gastrointestinal tract is significantly different from the female genital tract. Consequently, products made for the vagina may not be optimal for rectal use.

With the lack of effectiveness found in non-specific entry inhibitors such as cellulose sulfate [9], [10] and PRO 2000 [11], [12] and surfactants such as nonoxynol-9 [13]–[15], antiretroviral drugs are currently the lead candidates being pursued for topical microbicide development. Tenofovir (TFV) and UC781 are representative of nucleotide reverse transcriptase inhibitor (NRTI) and non-nucleoside reverse transcriptase inhibitor (NNRTI) drug classes, respectively. Both drugs have been formulated as vaginal microbicide aqueous gel products and have been evaluated in clinical trials [16]–[19]. Additionally, both vaginal gels have been evaluated clinically for rectal safety [20], [21]. While no safety concerns were noted in trial participants when either gel was used vaginally, some of the participants using the vaginal TFV 1% gel rectally exhibited gastrointestinal complications that included abdominal bloating, pain/cramps, and defecation urgency [20]. These symptoms are reminiscent of hyperosmolar enemas. Indeed, the vaginal TFV 1% gel was hyperosmolar and showed epithelial sloughing in ex vivo mucosal tissue [22]. These data reinforce the need for rectal-specific products – rectal microbicides (RMs) – specifically designed for use during RAI [23].

The use of ex vivo mucosal tissue to evaluate the safety and efficacy of HIV-1 prevention modalities has increased over the past decade. Several laboratories now utilize colonic tissue in addition to ectocervical tissue to test drug candidates and their formulations (topical microbicides) for safety and effectiveness [24]–[26]. Tissue is most often acquired as remainders from surgery [24]–[26], but colonic tissue also has been collected by flexible sigmoidoscopy (flex sig) [25]. The ability to use various sources of tissue for testing has the potential to increase the efficiency of product testing and reduce the time needed to acquire this information, thus streamlining the testing process. To effectively utilize flex sig tissue for microbicide testing, the tissue collected from both procedures needs to show consistency in results. There are several differences between resected and flex sig tissue that make comparing important and include (i) the anatomical location where the colon tissue is collected, (ii) the length of time between procedure and transport to the laboratory, (iii) pre-existing medical conditions and treatments, and (iv) the age of tissue donor. To demonstrate that tissue acquired by either procedure performs similarly in the ex vivo explant model, we compared the ability to infect the tissues with HIV-1 and also the safety and efficacy outcomes following exposure to novel rectal-specific antiretroviral microbicide products.

## Materials and Methods

### Human tissue

Surgically resected and flex sig tissues were acquired through approved protocols by the University of Pittsburgh (Pitt) Institutional Review Board (IRB). The Pitt IRB specifically approved both protocols: collection from patients undergoing surgery (IRB # PRO0602024) and collection from healthy individuals (IRB # PRO10070364). Normal human colonic tissue was acquired by surgical resection from persons undergoing surgery for non-inflammatory conditions (18 to 80 years old). The median age of the patients was 64 years old (range 35–75). The patients signed a general waiver for their surgical procedure which includes the use of their tissue for research purposes. After clearance from pathology, surgically resected tissue was collected by an “Honest Broker” whose role was to de-link identifiable patient information from the investigators. The Honest Broker also provided generalized demographic information, such as location of colon, age range, race, and gender of the patient. Flex sig tissues were collected from persons (18 to 65 years old) recruited for the study. The median age of the participants was 40 years old (range 19–61). These participants were healthy individuals with no known evidence of gastrointestinal disease. After signing a consent form, 20 biopsies were collected from the sigmoid colon, 15 cm from the anal margin using Jumbo forceps (Radial Jaw 4, Boston Scientific, Natick, MA) by a board-certified gastroenterologist. The flex sig tissue was provided with study identifiers de-linking participant information for the investigators. Regardless of procedure, tissue was placed in cold transport medium (L-15 supplemented with 10% fetal calf serum, 100 µg/ml streptomycin, 100 U/ml penicillin, 250 µg/ml amphotericin B, and 100 mM L-glutamine) and processed within 90 minutes.

### Rectal-specific microbicide products

#### Formulation preparation

Previously, four rectal specific placebo products, namely, aqueous gel, aqueous liquid, lipid gel and lipid liquid, were developed by our group [23]. These four products possessed a wide range of physical and chemical properties which could be utilized to formulate either a water soluble or a water insoluble drug candidate as a solution thereby providing a high concentration gradient for drug release. This panel of placebo products served as the basis for formulation of the hydrophilic drug (TFV, provided by CONRAD, Arlington, VA) and the lipophilic drug (UC781, provided by CONRAD).

TFV aqueous gel (1%) was prepared as a carbopol 974 (Lubrizol, Cleveland, OH) and sodium carboxymethylcellulose (Na CMC, high viscosity; Spectrum, New Brunswick, NJ) based formulation. Briefly, a solution of the preservatives (methylparaben and propylparaben) was prepared in purified water using heat (60–75°C). TFV (1%) was then dissolved in the solution by addition of NaOH (18% w/v) using an overhead propeller mixer (Model RW20, IKA Works, Inc. Wilmington, NC). Carbopol 974P (0.5%) and 1% Na CMC were added and mixed until dissolved. Then 0.1% disodium edetate and 2.5% glycerin were added and mixed to dissolve. Finally, NaOH was added to neutralize the carbopol for gel formation.

TFV aqueous liquid (1%) was formulated into a thermoreversible aqueous liquid. First, the preservative solution (methylparaben and propylparaben) was prepared at 60–75°C and then cooled to 8–10°C. Sodium citrate dehydrate NF (0.3%) (Spectrum), 2% glycerin (Spectrum) and 15% Poloxamer 407 (Pluronic F127; BASF Corporation, Mt. Olive, NJ) were added while stirring. TFV was added to the aqueous solution.

UC781 lipid gel (0.1%) was formulated into a lipid cream. Briefly, UC781, 0.1% vitamin E acetate (Spectrum) and 0.2% antioxidant butylated hydroxyanisole (Spectrum) were dissolved in a lipid solvent, 74.8% caprylic/capric triglyceride (Crodamol GTCC; Croda Inc., Edison, NJ). The lipid gelling agent, 24.8% glyceryl stearate and PEG-75 stearate (GelotTM 64; Gattefosse, Paramus, NJ) was then added into the lipid solvent containing UC781.

UC781 lipid liquid (0.1%) was formulated into a lipid liquid. Briefly, 94.8% isopropyl myristate (Crodamol IM; Croda) and 4.9% myristyl myristate (Crodamol MM; Croda) were heated to 60°C and stirred to dissolve using an overhead mixer. Antioxidant 0.2% butylated hydroxyanisole (Spectrum) and UC781 were added and mixed to uniformity.

#### Formulation characterization

Appearance: Formulations were evaluated by visual inspection for phase separation, color, clarity, consistency and particulates in clear, glass scintillation vials.

Drug content: TFV drug content was measured in triplicate using an established high performance liquid chromatography (HPLC) method. Briefly, a Dionex Ultimate 3000 system (Thermo Scientific, former Dionex, Bannockburn, IL) equipped with an autosampler, photodiode array detector, and Chromeleon version 6.8 software were used with an Acclaim 120 Å (4.6×150 mm, 3 µm) (Thermo Scientific, former Dionex) column. Separations were done in an isocratic system using a mixture of 10 mM K_2_HPO_4_ buffer solution containing 5 mM t-butylammonium bisulfate (pH 5.7) and methanol at a ratio of 90∶10. The flow rate was 1.0 ml/min. The wavelength was 260 nm. The linear range was from 1 to 200 µg/ml.

UC781 drug content was measured in triplicate using the same HPLC system as described above. The separation was achieved using a gradient method on an Acclaim 120 Å (4.6×150 mm, 3 µm) column. The initial mobile phase of acetonitrile:water at a ratio of 75∶25 was run for 10 min followed by a linear gradient over 30 min to a final mobile phase of acetonitrile:water at a ratio of 95∶5. The linear range was from 1 to 250 µg/ml.

Viscosity: Rheological profiles were determined using a cone and plate viscometer (Brookfield HADV III+ and LVDV III ultra, Brookfield Engineering Laboratories, Middleboro, MA). Shear stress over a range of shear rates from 0.1 rpm to 30 rpm was recorded and both increasing and decreasing curves generated at 25 and 37°C. All viscosities reported were apparent viscosities calculated as the ratio of shear stress to shear rate. For comparison purposes, viscosities were calculated at a fixed shear rate of 10 rpm.

pH: The pH of the TFV aqueous gel formulation was measured using a pH meter (Accumet AR 20, Fisher Scientific, Pittsburgh, PA) equipped with a flat surface pH electrode (Beckman Coulter Futura Flat Bulk Combination pH electrode, Fisher Scientific). The pH of the TFV aqueous fluid formulation was measured with the same instrument using a glass pH electrode (Accumet pH electrode, Fisher Scientific). The pH meter was calibrated with pH 4.0, 7.0 and 10.0 standards.

Osmolality: The osmolality of TFV aqueous formulations was measured using a vapor pressure osmometer (Model # 5520; Wescor, Inc, Logan, UT).

#### Short term stability

Studies were conducted for three months to evaluate product stability over the time frame required for colonic tissue evaluation. Gel formulations were packaged in glass, straight sided jars. Opaque jars were used for UC781 formulations to protect the product from light. Liquid formulations were packaged in glass Boston round bottles with poly-seal caps. Packaged product samples were stored at 25°C/60% RH and 40°C/75% RH. Testing at each stability time point included appearance, viscosity, and drug content for all samples. Additionally, pH and osmolality was tested for aqueous formulations.

#### Virus

HIV-1_BaL_ working stock was made by diluting a concentrated stock (Advanced Biotechnologies, Inc., Columbia, MD). Additionally, an infectious molecular clone of HIV-1_JR-CSF_ was obtained from the AIDS Research and Reference Reagent Program, DAIDS, NIAID, NIH. Stocks of the clone was made by transfecting 293T cells and collecting the 48 h supernatant. HIV-1_BaL_ and HIV-1_JR-CSF_ stocks were titrated in activated PBMCs using the Reed & Muench method [27]. Virus aliquots were stored at −80°C until used.

#### Polarized explants

The biopsies obtained from flex sig were variable in size, but overall similar to the explants made from surgical resections which were approximately 5 mm in diameter ×3 mm in depth. Polarized explant cultures were set-up as previously described [24]. Briefly, the tissue was placed with the luminal side up resting on medium-soaked gelfoam in a transwell. The edges around the explant were sealed with Matrigel (BD Biosciences, San Jose, CA). Tissues were cultured with the luminal surface at the air-liquid interface and 1 ml of medium (RPMI-1640 medium supplemented with 5% human AB serum, 100 U/ml IL-2 [Roche Applied Science, Indianapolis, IN], 100 µg/ml streptomycin, 100 U/ml penicillin, and 100 mM L-glutamine, 0.5 mg/ml Zosyn and 2.5 mM Hepes) in the basolateral compartment. For the first 24 h, the basolateral medium contained 5 µg/ml of phytohemagglutinin-P (Sigma-Aldrich, St. Louis, MO). Cultures were maintained at 37°C in a 5% CO_2_ atmosphere. Each study condition was assembled in duplicate. To compare the collection procedures on HIV-1 infection, titrations of virus were added to the apical surface either alone or with 50% normal, pooled human semen (Lee BioSolutions, Inc., St. Louis, MO). When the RM were tested, a 1∶5 dilution of the aqueous formulations (tenofovir 1% gel or liquid along with the corresponding placebo) or neat lipid formulations (UC781 lipid gel or liquid and the corresponding placebos) were mixed with the virus before adding to the apical surface. Tissues were cultured overnight, washed, and fresh culture medium replenished in the basolateral compartment. Every 3 to 4 days, basolateral supernatant was collected and fresh medium replaced. The collected supernatant was stored at −80°C. HIV-1 replication was monitored by p24 ELISA from the culture supernatants.

To investigate the possible impact of semen or rectal microbicide products on tissue viability, surgically resected or flex sig acquired colorectal tissues were placed in culture using our polarized protocol. Tissues were exposed to semen or RM overnight. As controls, tissue alone or tissue treated with a 1∶5 dilution of a 3% nonoxynol-9 containing gel (Gynol II, Revive Personal Products Company, Madison, NJ) were tested in parallel in the same tissue donor. The next day, tissues were washed and viability was evaluated using the MTT [1-(4,5-dimethylthiazol-2-yl)-3,5-diphenylformazan] assay and architecture assessed by histology [24].

#### Statistical analysis

Descriptive statistics were run on all variables of interest. For continuous variables, sample means and standard deviations were computed; for categorical variables, sample proportions were calculated. Medians and ranges were also included due to the non-normal nature of HIV-p24.

Linear mixed models were used to investigate the trajectory of ln(HIV-p24) over time and how it relates to colon location, HIV titration, effect of semen, and rectal microbicide efficacy. The natural log transformation of the outcome was chosen due to the highly right-skewed nature of the data.

For the colon location analysis, the linear mixed model included predictors for “day of culture”, colon location, their interaction, and a random effect to account for correlated observations from the same sample. The models for HIV titration and effect of semen each had predictors for “day of culture”, tissue collection method (surgically resected versus flex sig), their interaction, and a random effect. In addition, the models were run separately within each combination of virus (HIV-1_JR-CSF_ and HIV-1_BaL_) and titer (10^2^, 10^3^, and 10^4^). Finally, the linear mixed model for rectal microbicide efficacy includes predictors for “day of culture”, microbicide type (control versus microbicide versus placebo version of microbicide), their interaction, and a random effect. The microbicides (TFV aqueous gel, TFV aqueous fluid, UC781 lipid gel, and UC781 lipid liquid) and placebos were analyzed separately for each of the flex sig and surgical resection methods. Of primary interest in these statistical models was whether there was a significant difference in p24 values across time (significant interaction). If not, main effects of colon location, tissue collection method, or rectal microbicide efficacy were investigated. Changes in tissue viability (MTT assay) were determined by a Mann-Whitney test (semen) or an ANOVA with Bonferroni’s Multiple Comparison Test (product).

Each of the analyses was conducted at the 5% significance level with no adjustments for multiplicity. All analysis was conducted in SAS (Cary, NC) as well as GraphPad (GraphPad Prism 5, version 5.04).

## Results

### HIV-1 infection in surgically resected tissue

**Figure 1:**
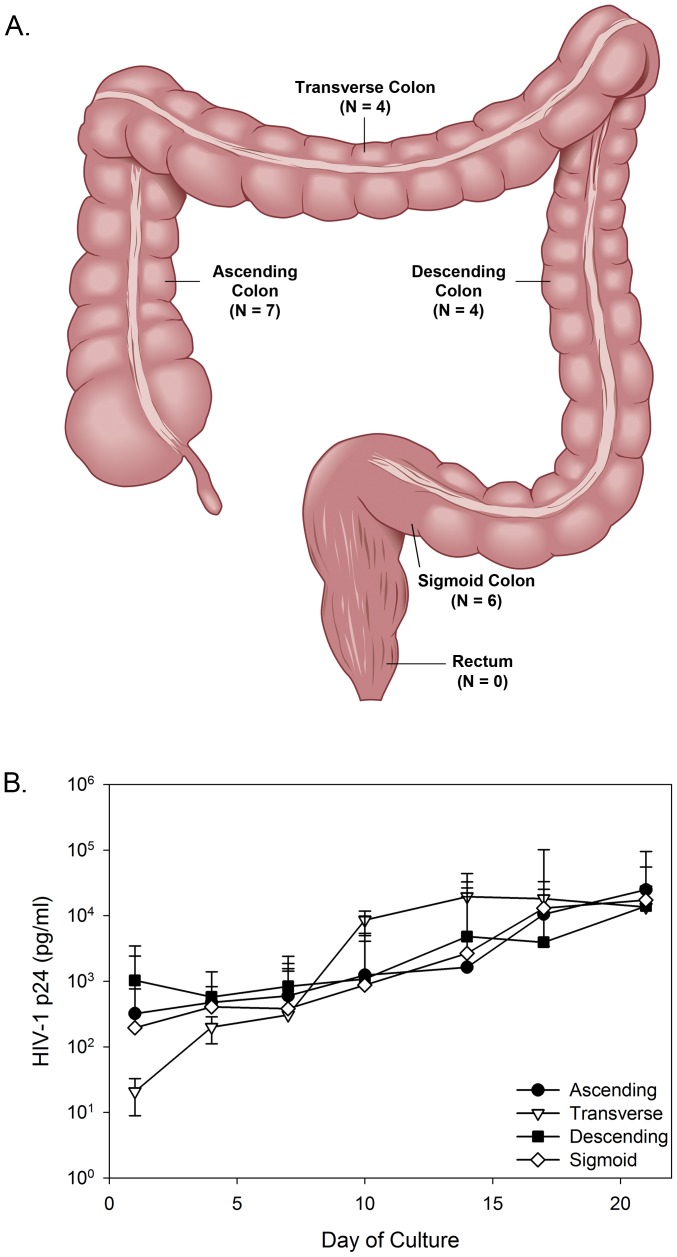
HIV-1 replication across four major areas of the colon. Tissues from the ascending, transverse, descending, and sigmoid colon were evaluated for the ability of HIV-1_BaL_ to infect and replicate. Tissues were set-up in polarized conditions and HIV-1 was applied to the apical, luminal surface. After overnight culture, the tissues were washed and followed through day 21 of culture. HIV-1 replication (p24 ELISA) was monitored in the basolateral supernatant collected every 3 to 4 days of culture. The data presented are a median of 7, 6 (ascending and sigmoid), and 4 (transverse and descending) tissues ±95% confidence interval.

Throughout the colon are isolated lymphoid follicles which serve as inductive sites for immune responses [28]. The number of follicles generally increases toward the anus with the greatest numbers found in the rectum [29], [30]. Consequently, there may be differences in HIV-1 replication across the colon. While several laboratories use surgically resected tissue, none have investigated potential differences in the ability to infect distinct areas of the colon with HIV-1. Therefore, HIV-1 replication kinetics were evaluated across the colon: ascending, transverse, descending, and sigmoid (Fig. 1A). To note, no surgically resected rectal tissue was obtained in our study. Colonic tissues were from patients (age ranges 30 to 79 years old). The number of transverse and descending tissues (n = 4 each) available to compare were limited. Overall, similar replication kinetics and magnitude of viral replication were noted from the different locations of the colon (P = .56) (Fig. 1B). Thus, location of tissue does not appear to restrict HIV-1 infection and replication in surgically resected tissue.

### Comparing HIV-1 infection in surgically resected with flex sig tissues

**Figure 2:**
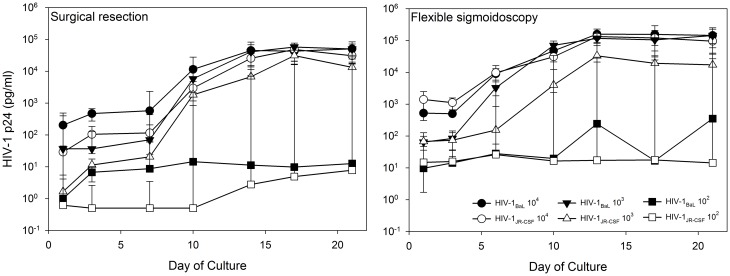
HIV-1 replication in colonic tissue obtained through surgical resection and flexible sigmoidoscopy. Polarized cultures were set-up in duplicate. Titrations of HIV-1_BaL_ and HIV-1_JR-CSF_ were applied to the apical surface and allowed to culture overnight. The following day, the tissues were thoroughly washed and followed through day 21 of culture. HIV-1 replication (p24 ELISA) was monitored in the basolateral supernatant collected every 3 to 4 days of culture. The data presented are the median ±95% confidence interval of 7 tissues for HIV-1_BaL_ and 5 tissues for HIV-1_JR-CSF_ for surgical resections; and 12 tissues for HIV-1_BaL_ and 9 tissues for HIV-1_JR-CSF_ for flexible sigmoidoscopy.

Tissue obtained from surgical resections is from donors that are generally older than participants recruited for collection of flex sig tissue. Moreover, surgically resected tissue is collected from patients with medical conditions that may require treatment (for example chemotherapy for cancer) and have a more prolonged ischemic time (up to 3 hours) due to the required release from the pathology department while flex sig tissue is collected immediately from healthy participants. These differences led us to compare HIV-1 replication kinetics between these tissue collection procedures. Surgically resected and flex sig tissues were exposed to titrations of HIV-1_BaL_ and HIV-1_JR-CSF_ (Fig. 2). Surgically resected tissue replicated 10^4^ TCID_50_ of HIV-1_BaL_ to 4.7 log_10_ p24. This was similar to the 10^3^ TCID_50_ (4.8 log_10_) and >1000-fold better than the 10^2^ TCID_50_ of HIV-1_BaL_ (1.2 log_10_). Flex sig tissue replicated the 10^4^ and 10^3^ TCID_50_ of HIV-1_BaL_ to 5.2 log_10_ p24, while 10^2^ TCID_50_ of HIV-1_BaL_ replicated to 2.5 log_10_ p24. The statistical model showed there were no differences in p24 trajectory between the tissue collection procedures at any of the titrations (10^4^: P = .7704; 10^3^: P = .5123; 10^2^: P = .6216). As compared to HIV-1_BaL_, HIV-1_JR-CSF_ is a T-cell tropic R5-using virus and may better represent virus that is sexually transmitted [31], [32]. The peak HIV-1_JR-CSF_ p24 for 10^4^, 10^3^ and 10^2^ TCID_50_ (respectively) was 4.7, 4.5, and 0.9 log_10_ p24 for surgically resected tissue compared to 5.1, 4.5, and 1.4 log_10_ p24 for flex sig tissue. While the kinetics of replication for both viruses were similar, flex sig tissue replicated HIV-1_JR-CSF_ better than surgically resected tissue at all titrations of virus (10^4^: P = .0020; 10^3^: P = .0355; 10^2^: P = .0363). In general, the 10^2^ TCID_50_ of either virus resulted in highly variable replication, confirming this dose of HIV-1 was beyond the limit of reproducible infection.

**Figure 3:**
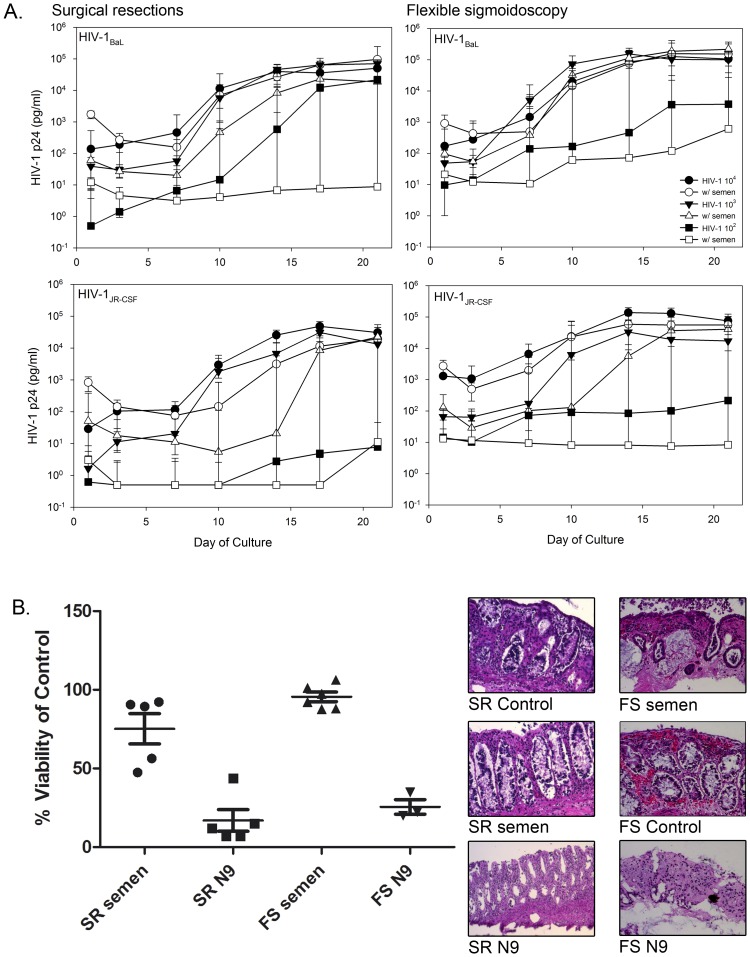
The impact of semen on HIV-1 replication by colonic tissue. Tissues were set-up in duplicate in polarized cultures to evaluate the effects of semen on HIV-1 infection (A) or on tissue viability/architecture (B). (A) HIV-1_BaL_ or HIV-1_JR-CSF_ was mixed with 50% whole semen and applied to the apical surface and cultured overnight. The following day, the tissues were thoroughly washed and followed through day 21 of culture. HIV-1 replication (p24 ELISA) was monitored in the basolateral supernatant collected every 3 to 4 days of culture. The data presented are the median ±95% confidence interval of 5 tissues for HIV-1_BaL_ and HIV-1_JR-CSF_ each for surgical resections; and 7 tissues for HIV-1_BaL_ and 5 tissues for HIV-1_JR-CSF_ for flexible sigmoidoscopy. (B) 50% semen was added to the apical surface of surgical resections (SR) or flexible sigmoidoscopy (FS). As controls, medium alone (untreated control) or medium containing a 1∶5 dilution of a 3% nonoxynol-9 (N9)-containing gel were used. After an overnight culture, tissues were washed with one piece further cultured in medium containing 1-(4,5-dimethylthiazol-2-yl)-3,5-diphenylformazan for the MTT assay or the other piece fixed in paraformaldehyde for hematoxylin and eosin staining. The MTT assay results are presented as scatter plots with the horizontal lines denoting the mean ± standard deviation of 5 independent tissues. The histology is representative of one of those tissues.

Because HIV-1 infection occurs primarily through sex, HIV-1 infection was evaluated in the presence of semen. Surgically resected and flex sig tissues were exposed to titrations of HIV-1 in the absence or presence of 50% whole semen (Fig. 3A). Overall, HIV-1_BaL_ and HIV-1_JR-CSF_ replication was reduced in the presence of semen regardless of tissue source. HIV-1_BaL_ at 10^3^ TCID_50_ and HIV-1_JR-CSF_ at 10^4^ and 10^3^ TCID_50_ with semen exhibited delays in virus replication, but by day 21 the cultures had similar p24 levels as tissue exposed to HIV-1 in the absence of semen. For both viruses at 10^2^ TCID_50_, viral replication was reduced in both tissues in the presence of semen. Similar to HIV titration without semen, the p24 trajectories in HIV-1_BaL_ did not differ between collection procedures (10^4^: P = .5062; 10^3^: P = .2936; 10^2^: P = .1426), but flex sig tissues had consistently greater viral replication for 10^3^ and 10^2^ TCID_50_, regardless of day (10^4^: P = .4862; 10^3^: P = .0460; 10^2^: P = .0050). For HIV-1_JR-CSF_, p24 trajectories did differ for 10^4^ TCID_50_ (P = .0340), but further investigation found no differences at any day of culture. Overall, for the 10^2^ TCID_50_ titrations, flex sig had greater viral replication than surgically resected tissue independent of semen across days (HIV-1_BaL_: P = .0050; HIV-1_JR-CSF_: P = 0.0110). The lack of enhancement in HIV-1 replication by semen could not be attributed to loss of tissue viability as both surgically resected and flex sig tissues retained mitochondrial activity, as determined by the MTT assay, and showed retention of their architecture (Fig. 3B).

### Development and characterization of rectal-specific microbicide formulations

These data show reproducible HIV-1 infection from the four major areas of the colon and between surgically resected and flex sig tissue. Therefore, use of either tissue should demonstrate comparable data when evaluating RMs. To test our hypothesis, four rectal-specific microbicide formulations were developed representing either a gel or liquid dosage form containing TFV or UC781. These four products possess distinct physicochemical properties representing a wide range for rectal-specific microbicide products (Table 1). Given the hydrophilic nature of TFV, TFV formulations were aqueous-based. Conversely, based on the hydrophobicity of UC781, those formulations were of a lipid nature. The TFV aqueous gel exhibited shear thinning and non-Newtonian pseudoplastic behavior. In a shear thinning system, viscosity decreases as the applied shear to the product is increased and a non-Newtonian fluid exhibits variable viscosity over a range of shear rates. The TFV aqueous liquid utilized Poloxamer to create a thermoreversible product and exhibited Newtonian rheological profile at 25°C with no viscosity change over a range of shear rates. However, it exhibited shear thinning non-Newtonian pseudoplastic rheological profile at 37°C. The rheological profile of the UC781 lipid gel was shown to be shear thinning, non-Newtonian and thixotropic with a lower viscosity at 37°C. In a thixotropic system, the viscosity decrease observed as increased shear is applied requires time to return to original values as the shear is removed. In contrast, the UC781 lipid liquid was Newtonian, showing constant viscosity over a range of shear rates with similar viscosities observed at 25°C and 37°C. The pH of TFV aqueous gel and liquid were 7.0, which approximates the physiological pH of the rectal compartment [33]. The TFV aqueous gel and liquid were 479 mmol/kg and 593 mmol/kg, respectively, indicating they were <2-fold above normal physiologic osmolalities (∼300 mmol/kg). The stability data generated for all formulations at 25°C/60% RH and 40°C/75% showed all products to remain within target specifications (Table 2). Stability data demonstrated product integrity for the time frame required for colonic tissue explant evaluation.

**Table 1:** Physiochemical characterization of the rectal-specific microbicide formulations.

Formulation	Appearance	Drug content^a^(%, w/w)	Rheological profile	Viscosity (cp)		pH	Osmolality^a^(mmol/kg)
				25°C	37°C		
Tenofovir aqueous gel	Slightly hazy, colorless gel	0.971±0.026	Shear thinning, non-Newtonian	3049	2646	7.0	479±27
Tenofovir aqueous liquid	Clear, colorless, easily spreadableliquid at 25°C, clear, colorless gel at 37°C	0.993±0.022	Newtonian at 25°C, shear thinningat 37°C	101	1781	7.0	593±16
UC781 lipid gel	yellowish creamy semisolid	0.096±0.001	Shear thinning, thixotropic	4329	1667	–*	–
UC781 lipid liquid	Yellow, easily spreadable liquid	0.096±0.002	Newtonian	5.3	3.8	–	–

*not required.

a Data are presented as the average ± standard deviation of three independent tests.

**Table 2:** Stability of rectal-specific microbicide formulations.

Formulation	Condition/Interval (month)		Drug content (%, w/w)	Viscosity (cp)	pH	Osmolality (mmol/kg)
Tenofovir aqueous gel	25°C	Initial	0.971±0.026	3050	7.0	479
		1	0.972±0.004	3293	6.9	461
		2	0.983±0.008	3407	7.0	512
		3	0.999±0.007	3247	7.1	607
	40°C	1	0.989±0.017	3205	7.0	474
		2	1.002±0.012	3086	7.0	508
		3	0.984±0.036	2946	7.0	615
Tenofovir aqueous liquid	25°C	Initial	0.993±0.022	101	7.0	593
		1	0.964±0.059	103	7.1	585
		2	1.002±0.010	116	7.0	570
		3	0.993±0.021	108	6.9	676
	40°C	1	0.978±0.056	109	7.0	555
		2	0.999±0.035	116	6.9	613
		3	0.985±0.015	112	7.0	495
UC781 lipid gel	25°C	Initial	0.096±0.001	4329	–*	–
		1	0.093±0.001	4122	–	–
		2	0.091±0.001	4256	–	–
		3	0.091±0.002	4529	–	–
	40°C	1	0.093±0.001	4246	–	–
		2	0.093±0.001	4298	–	–
		3	0.089±0.002	4445	–	–
UC781 lipid liquid	25°C	Initial	0.096±0.002	5.3	–	–
		1	0.096±0.001	5.3	–	–
		2	0.094±0.003	5.2	–	–
		3	0.095±0.001	5.3	–	–
	40°C	1	0.094±0.001	5.3	–	–
		2	0.093±0.002	5.2	–	–
		3	0.092±0.003	5.3	–	–

*not required.

### Rectal-specific microbicide evaluation for safety and efficacy against HIV-1

**Figure 4:**
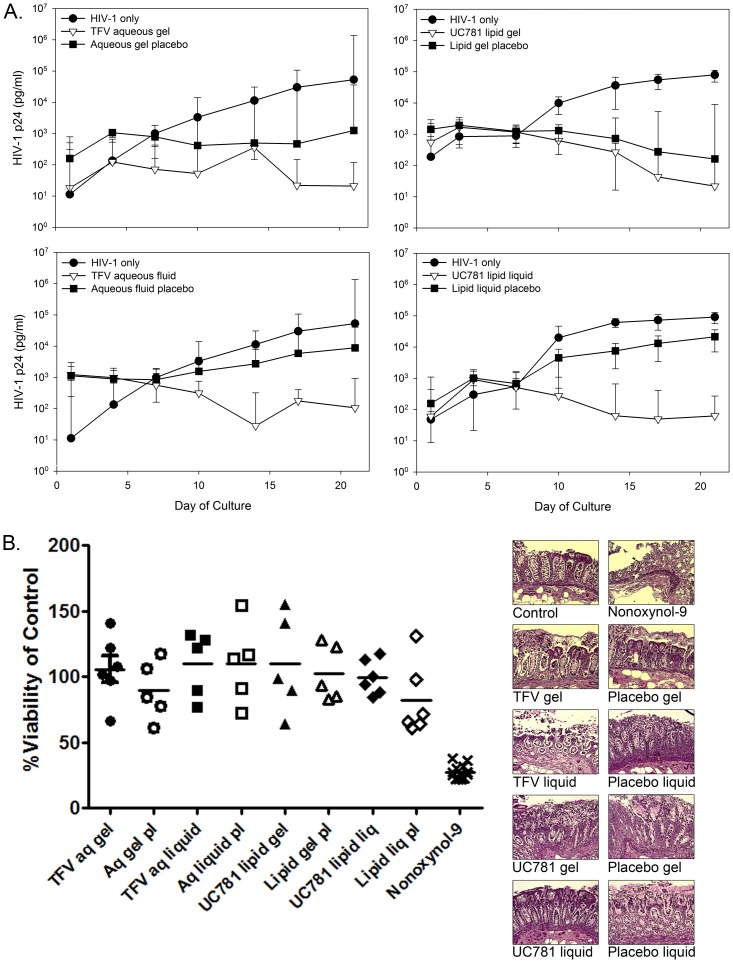
Rectal-specific microbicide (RM) products protect surgically resected colonic tissue from HIV-1 infection and are safe. Colonic tissue obtained through surgical resections was used to create polarized explant cultures. The explants were set-up in duplicated. (A) Efficacy: Tenofovir (TFV) aqueous-based RMs were diluted 1∶5 in medium with HIV-1_BaL_ and applied to the apical surface. The UC781 lipid-based RMs were used neat and mixed with HIV-1_BaL_, and then applied to the apical surface. The corresponding placebo products were handled in a similar fashion. After an overnight culture, the tissues were thoroughly washed and followed through day 21 of culture. HIV-1 replication (p24 ELISA) was monitored in the basolateral supernatant collected every 3 to 4 days of culture. The data presented are the median ±95% confidence interval of 5 independent tissues for each treatment. (B) Tissue viability: After an overnight culture with the indicated RM product, medium alone (untreated control) or medium containing a 1∶5 dilution of a 3% nonoxynol-9 (N9)-containing gel, tissues were washed with one piece further cultured in medium containing 1-(4,5-dimethylthiazol-2-yl)-3,5-diphenylformazan for the MTT assay or the other piece fixed in paraformaldehyde for hematoxylin and eosin staining. The MTT assay results are presented as scatter plots with the horizontal lines denoting the mean ± standard deviation of 5 independent tissues. The histology is representative of one of those tissues.

The TFV and UC781 formulations were evaluated using surgically resected and flex sig tissue for the ability to block HIV-1 infection and their safety in mucosal tissue. In surgically resected tissue, there was a >2 log_10_ reduction of p24 between the active rectal microbicide formulations compared to the HIV-1 only treated tissues (Fig. 4A). The TFV and UC781 formulations all provided protection against HIV-1 infection as shown by a significant separation of HIV-1 p24 (P≤.05) by day 10 of culture. The aqueous and lipid gel placebos both showed a partial reduction of HIV-1 infection in the tissue, but none of the placebos significantly impacted HIV-1 infection of the tissue (P>.05). The MTT assay and histology showed both formulations of TFV and UC781 retained surgically resected colonic tissue viability and maintained tissue architecture (Fig. 4B). Nonoxynol-9 treatment (toxicity control) resulted in a significant reduction in tissue viability compared to the untreated tissue (P≤.01). Histologically, the tissue was stripped of the epithelium and the lamina propria was acellular compared to the control tissue.

**Figure 5:**
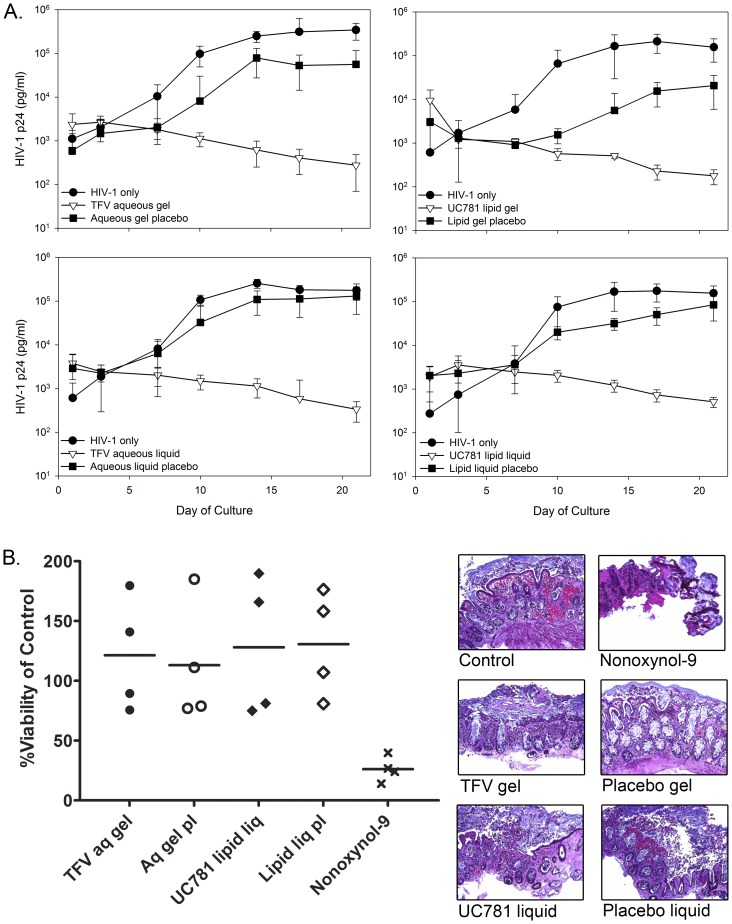
Rectal-specific microbicide (RM) products protect colonic tissue obtained through flexible sigmoidoscopy from HIV-1 infection and are safe. Colonic tissue obtained through flexible sigmoidoscopy was used to create polarized explant cultures. The explants were set-up in duplicate. (A) Efficacy: Tenofovir (TFV) aqueous-based RMs were diluted 1∶5 in medium with HIV-1_BaL_ and applied to the apical surface. The UC781 lipid-based RMs were used neat and mixed with HIV-1_BaL_, and then applied to the apical surface. The corresponding placebo products were handled in a similar fashion. After an overnight culture, the tissues were thoroughly washed and followed through day 21 of culture. HIV-1 replication (p24 ELISA) was monitored in the basolateral supernatant collected every 3 to 4 days of culture. The data presented are the median ±95% confidence interval of 3 to 5 independent donors for each treatment. (B) Tissue viability: After an overnight culture with the indicated RM product, medium alone (untreated control) or medium containing a 1∶5 dilution of a 3% nonoxynol-9 (N9)-containing gel, tissues were washed with one piece further cultured in medium containing 1-(4,5-dimethylthiazol-2-yl)-3,5-diphenylformazan for the MTT assay or the other piece fixed in paraformaldehyde for hematoxylin and eosin staining. The MTT assay results are presented as scatter plots with the horizontal lines denoting the mean ± standard deviation of 4 independent donors. The histology is representative of one of those donors.

Using flex sig biopsies, consistent efficacy results were found when compared to the surgically resected tissue. A >2.5 log_10_ reduction of p24 was noted from the TFV and UC781 treated tissues to HIV-1 only tissues (Fig. 5A). The TFV and UC781 formulations all provided protection against HIV-1 infection as shown by a significant separation of HIV-1 p24 (P≤.05) by day 10 of culture. While a decrease in p24 was noted for tissue treated with the placebo gels and liquids, none were significantly different from the HIV-1 control tissues (P>.05). Safety testing was performed on the TFV aqueous gel and UC781 lipid liquid along with their respective placebos (Fig. 5B). Flex sig tissue viability was retained after exposure to the active and placebo formulations. Nonoxynol-9 significantly decreased tissue viability (P<.05). Histologically, the formulations showed no impact on epithelial integrity while the nonoxynol-9-treated tissue showed stripping of the epithelium and necrosis of the lamina propria. These data are consistent with the findings from the safety testing using surgically resected tissue (Fig. 4B).

## Discussion

Because the rectum and lower gastrointestinal tract are structurally and functionally different from the vaginal vault, RM design has had two different approaches to establishing an effective rectally administered microbicide [23]. The first approach creates a formulation that spreads and coats all surfaces of the rectum and distal colon (liquid) rapidly prior to RAI and exposure to infected secretions. The second approach creates a deformable, erodible barrier that would remain localized at the administration site (semi-solid gel). These formulations must allow drugs to be solubilized to permit a pharmacological response. We evaluated the formulation of two drug candidates; one hydrophilic (TFV) and the other hydrophobic (UC781), using colonic tissue collected via different methods. The differing chemical nature of these two drug candidates permitted study of aqueous- and lipid-based products formulated into both liquids and gels. The rheological profile of a rectal product dictates its behavior with respect to mucosal coating and product erosion following product administration. The liquid products were Newtonian with respect to their flow behavior irrespective of whether they were aqueous or lipid based. Such a product would exhibit no change in viscosity with increased shear force. Since it is intended that liquid products would be “enema-like” and deliver drug further into the rectal compartment, a Newtonian product would easily spread over the mucosal surface. Conversely the gel products exhibited non-Newtonian behavior. Such a product requires introduction of shear to induce flow. Hence, the gel products would form an erodible barrier in which the product would remain localized and shear forces such as sexual intercourse would facilitate spreading. Developing both product types allowed for study of differing product distribution profiles to deliver TFV and UC781 to areas that would be exposed to potential infectious semen. Both TFV and UC781 RM formulations prevented HIV-1 infection in mucosal tissue, regardless of tissue source, and retained tissue viability. These data support further clinical testing of these RM products. It is hoped that rectal-specific formulations will be well tolerated in the vaginal compartment because the ideal microbicide formulation should have dual compartment safety and effectiveness.

Topical microbicide evaluation relies on a testing algorithm to define product activity and safety [22], [34], [35]. More researchers are now incorporating mucosal tissue for product evaluation into their algorithms. The ex vivo mucosal tissue typically has been acquired from surgical resections. For colonic tissue, surgical resections are often obtained from an older population who has undergone treatment for colorectal cancer. Acquisition of this tissue is dependent on an opportunistic surgical schedule and clearance from pathology. This makes tissue procurement unpredictable and prolongs the ischemic time (time from surgery to placement into transport medium). These challenges can be overcome by using a younger, healthier population of participants to obtain flex sig biopsies which may more accurately reflect persons who would be the primary users of topical microbicides. Despite requiring active recruitment, flex sig tissue allows for immediate retrieval of tissue from pre-scheduled appointments. The virus that is sexually transmitted uses the CCR5 co-receptor. Therefore, the HIV-1 traditionally used to infect mucosal tissue for topical microbicide evaluation has been HIV-1_BaL_ likely because it was easily accessible and one of the few viral isolates at the time that used CCR5 [22], [24], [25]. HIV-1_BaL_ was isolated from infected macrophages [36] and has been propagated in this cell type for almost 30 years. Other viruses including primary viral isolates and CXCR4-using HIV-1 have been used for infection of mucosal tissue [24], [37] and show similar or slightly better replication as compared to HIV-1_BaL_. In this study, HIV-1 infection was compared between tissue types using HIV-1_BaL_ and HIV-1_JR-CSF_. HIV-1_JR-CSF_ is a primary isolate (molecular clone) with a predilection for infecting T cells as compared to macrophage [32]. This may better represent the virus that is sexually transmitted as the founder population of infected cells is likely T cells [38], [39]. Infection of surgically resected and flex sig tissue showed similar replication kinetics for both viruses. However, the magnitude of HIV-1 replication in flex sig tissue was greater as compared to the surgically resected tissue by 0.4 log_10_ at the highest virus titer and 2 log_10_ at the lowest virus titer. While the magnitude of viral replication was significantly better for flex sig tissue, overall our findings show similar trends between surgically resected and flex sig tissues for HIV-1 infection and inhibition by rectal-specific microbicide formulations. These data show surgically resected and flex sig colonic tissue, while not identical, can be infected with HIV and should allow for greater options in testing microbicide products.

Typically semen delivers HIV-1 to the mucosa. Semen is a bioactive secretion containing immune cells, soluble factors, and other molecules [40]–[48] that have been shown to inhibit or enhance HIV-1 infection ex vivo independently of the pH [49], [50]. Using whole semen at a 50% concentration did not enhance HIV-1 infection for either surgically resected or flex sig tissue. We chose to use 50% whole, pooled semen because whole semen normally will be present during coitus and likely modestly diluted in other mucosal secretions (cervicovaginal and rectal fluids). Semen includes several soluble factors; one of them being IL-7, which is a central cytokine for T cell homeostasis [51]. Our culture incorporates IL-2 in the medium to support T cell viability so the additional effect of IL-7 on HIV-1 replication [47] may not be apparent in our model. Semen enhancing viral infection (SEVI) was shown to increase HIV-1 infection/replication in tonsil tissue when HIV-1 was applied at a suboptimal dose [43]. In our model, 10^2^ TCID_50_ of HIV-1_BaL_ and HIV-1_JR-CSF_ infects tissue inconsistently. When HIV-1 was applied in the presence of 50% semen, no increase in HIV-1 infection/replication or improved consistency of infection was found despite showing retention of tissue viability. Moreover pooled semen was used in the experiments presented here to reduce donor variability, thus the SEVI activity, attributed to amyloid fibrils formed by the prostatic acidic phosphatase fragments, is likely diminished because it has been shown to be donor specific [45]. Thus the competing inhibiting [40], [42], [46], [48] and enhancing [41], [43], [45], [47] activity of whole semen may have a net neutral effect on HIV-1 infection in mucosal tissue ex vivo.

The primary reason for evaluating surgically resected and flex sig tissues is to incorporate either tissue source interchangeably for rectal microbicide evaluation. To this end, novel RM formulations of TFV and UC781 were evaluated and compared in both tissue types. These formulations represent very different products, aqueous and lipid, with different rheological properties. As compared to topical microbicides that were designed for vaginal use, these products have a neutral pH (aqueous formulations only) and are slightly above isoosmolar [23]. Hyperosmolar gels when used rectally have been shown to strip the colonic epithelium and result in bowel urgency and abdominal bloating [20], [52]. These adverse events could lead to lack of product use. Further, in vitro testing showed that hyperosmolar microbicides [22] and lubricants [53] induced epithelial sloughing of the colonic epithelium and negligible loss in tissue viability. This gives some credence that the ex vivo model may reflect actual product use. The rectal-specific microbicides evaluated here showed retention of viability and architecture by both tissue sources. This likely reflects the near isotonic nature of the formulations. Both TFV and UC781 formulations prevented HIV-1 infection of surgically resected and flex sig tissues. This is the first time flex sig tissue has been used to evaluate topical microbicides ex vivo. Collectively, our data suggest these rectal-specific topical microbicide formulations should be safe and could reduce the acquisition of HIV-1 infection when used properly. Further testing is warranted to fully ascertain the safety and potential efficacy in early phase 1 clinical trials that incorporate the ex vivo challenge assay to provide linkages between drug distribution (pharmacokinetics) and drug activity (pharmacodynamics) [20], [21].

Because mucosal tissue contains HIV-1 target cells in physiologically relevant ratios, it provides an integrated model for understanding HIV-1 infection and product testing [54]. However, there are limitations [54], [55] in the use of mucosal tissue which include: i) loss of mucus and flora, ii) absence of blood supply with loss of hormonal control and capacity to recruit immune cells, and iii) loss of tissue architecture after ∼36h [24], [25]. Knowing these obstacles, mucosal tissue obtained from surgically resected and flex sig procedures provided comparable results in terms of HIV-1 infection, responses to semen, and the ability of RM formulations to prevent HIV-1 infection. The use of tissue collected from surgical resections or flex sig allows expansion of HIV-1 prevention product testing.
